# Receptor-binding domain of SARS-CoV-2 is a functional αv-integrin agonist

**DOI:** 10.1016/j.jbc.2023.102922

**Published:** 2023-01-18

**Authors:** Emma G. Norris, Xuan Sabrina Pan, Denise C. Hocking

**Affiliations:** 1 Department of Pharmacology and Physiology, University of Rochester School of Medicine and Dentistry, Rochester, New York, USA; 2 Department of Biomedical Engineering, University of Rochester School of Medicine and Dentistry, Rochester, New York, USA

**Keywords:** SARS-CoV-2, Receptor binding domain, Fibronectin, Extracellular matrix protein, Integrin, Cell adhesion, Focal adhesions, ACE2, angiotensin-converting enzyme 2, CoV, coronavirus, ECM, extracellular matrix, FAK, focal adhesion kinase, FN-null MEF, fibronectin-null mouse embryonic fibroblast, GST, glutathione S-transferase, RBD, receptor-binding domain, RGD, Arg-Gly-Asp, SAEC, small airway epithelial cell, SARS, severe acute respiratory syndrome, SPR, surface plasmon resonance

## Abstract

Among the novel mutations distinguishing SARS-CoV-2 from similar coronaviruses is a K403R substitution in the receptor-binding domain (RBD) of the viral spike (S) protein within its S1 region. This amino acid substitution occurs near the angiotensin-converting enzyme 2–binding interface and gives rise to a canonical RGD adhesion motif that is often found in native extracellular matrix proteins, including fibronectin. Here, the ability of recombinant S1-RBD to bind to cell surface integrins and trigger downstream signaling pathways was assessed and compared with RGD-containing, integrin-binding fragments of fibronectin. We determined that S1-RBD supported adhesion of fibronectin-null mouse embryonic fibroblasts as well as primary human small airway epithelial cells, while RBD-coated microparticles attached to epithelial monolayers in a cation-dependent manner. Cell adhesion to S1-RBD was RGD dependent and inhibited by blocking antibodies against α_v_ and β_3_ but not α_5_ or β_1_ integrins. Similarly, we observed direct binding of S1-RBD to recombinant human α_v_β_3_ and α_v_β_6_ integrins, but not α_5_β_1_ integrins, using surface plasmon resonance. S1-RBD adhesion initiated cell spreading, focal adhesion formation, and actin stress fiber organization to a similar extent as fibronectin. Moreover, S1-RBD stimulated tyrosine phosphorylation of the adhesion mediators FAK, Src, and paxillin; triggered Akt activation; and supported cell proliferation. Thus, the RGD sequence of S1-RBD can function as an α_v_-selective integrin agonist. This study provides evidence that cell surface α_v_-containing integrins can respond functionally to spike protein and raises the possibility that S1-mediated dysregulation of extracellular matrix dynamics may contribute to the pathogenesis and/or post-acute sequelae of SARS-CoV-2 infection.

Coronaviruses (CoVs) are a diverse group of positive-stranded RNA viruses named for the distinctive crown-like protrusions on their surfaces. CoVs can infect a wide range of mammalian and avian species, causing mild to severe respiratory infections (1). At present, seven different CoVs are known to infect humans, four of which cause only mild disease (2). Within the past 20 years, three CoVs have emerged that are capable of causing more severe disease in humans: SARS-CoV-1, the cause of severe acute respiratory syndrome (SARS); MERS-CoV, the cause of Middle East respiratory syndrome (MERS); and SARS-CoV-2, the cause of COVID-19 (1). The most common symptoms of COVID-19 infection are fever, cough, shortness of breath, and fatigue (3), but disease progression varies widely with approximately 20% of nonvaccinated patients experiencing severe acute disease (4). Acute respiratory distress syndrome (5), as well as myocardial (6), renal (7), hepatic (8),and digestive (9) complications have all been reported. In addition, over half of patients with COVID-19, including those with mild, acute symptoms, exhibit a range of short and long-term post-acute sequelae that include pulmonary abnormalities, functional mobility impairment, fatigue, and joint pain (10, 11). The complex clinical manifestations of acute and post-acute COVID-19 suggest a dysregulated host response to infection that triggers immunoinflammatory, thrombotic, and parenchymal disorders (12), Yet, the pathophysiological mechanisms responsible for the diverse disease phenotypes remain largely unknown.

The extracellular matrix (ECM) glycoprotein, fibronectin, is an essential regulator of connective tissue homeostasis (13), epithelial morphogenesis (14), endothelial barrier maintenance (15, 16), local arteriolar tone (17, 18), and tissue repair (19). Fibronectin also serves a significant role in host–pathogen interactions, as fibronectin-binding and fibronectin-mimicking proteins have been identified across a broad spectrum of microbial pathogens (20). Compared with SARS-CoV-1, the spike (S)1 subunit of SARS-CoV-2 contains a novel mutation that mimics a bioactive sequence in fibronectin: a Lys (K) to Arg (R) mutation in the receptor-binding domain (RBD), resulting in the adhesive Arg-Gly-Asp (RGD) motif of fibronectin’s integrin-binding domain (21). In fibronectin, the RGD sequence is located in a short loop that extends from the tenth type III repeat (FNIII10) where it mediates adhesion for a variety of cell types, including epithelial cells, endothelial cells, and fibroblasts, *via* β_1_ and β_3_ integrins (22, 23). Ligation of cell surface integrins with the RGD sequence of fibronectin triggers a cascade of cell signaling events, including protein kinase C activation and Rho-mediated actomyosin contractility, that lead to changes in cell shape (24), focal adhesion composition (25, 26), and extracellular matrix assembly (27). Critically, activation of components of these adhesion-based signaling cascades has been associated with reduced endothelial and epithelial barrier function and increased prevalence of inflammatory diseases (28).

SARS-CoV-2 infects epithelial cells of both the respiratory (29) and gastrointestinal tracts (30) *via* S1-mediated recognition of angiotensin-converting enzyme 2 (ACE2) on host cell surfaces (31). Initial evaluation of interresidue distances within the crystal structure of SARS-CoV-2 spike in complex with ACE2 suggested that the RGD motif of S1 is located adjacent to, but not included within, the ACE2-binding surface (32). More recent analysis indicates that Arg403 of S1 is highly conserved across SARS-CoV-2 lineages and may facilitate viral engagement of human cells *via* an ionic interaction with residue Glu37 of ACE2 (33). Positive detection of S1-integrin binding *via* solid-phase ELISA assays has been reported by several independent groups for both α_5_β_1_ (34, 35) and α_v_β_3_ (36) integrins. Viral infection studies further showed that cell-surface binding and viral uptake of SARS-CoV-2 can be inhibited by integrin antagonists, including the peptide inhibitors Cilengitide (36) and ATN-161 (34, 37), as well as by cell-permeable inhibitors of inside-out integrin signaling (38). Thus, converging evidence suggests that S1-integrin interactions occur during SARS-CoV-2 infection, although the specificity and selectivity for specific integrins, as well as the implications for SARS-CoV-2 infection and disease remain to be elucidated. In the present study, we investigated S1-integrin interactions using both primary human small airway epithelial cells, as well as fibronectin-null mouse embryonic fibroblasts (FN-null MEFs). FN-null MEFs do not produce fibronectin, laminin, or vitronectin (39, 40) and are cultured in the absence of serum, allowing for the characterization of S1 binding to cell surface receptors and the identification of intracellular signals triggered by S1-integrin engagement without interference from other adhesive ligands (40, 41, 42). Results of this study indicate that the RGD motif contained within S1 is a cryptic, low-affinity, α_v_ integrin ligand that can mediate cell adhesion, spreading, and proliferation to a similar extent as native fibronectin. RBD-integrin engagement triggers canonical integrin-mediated signaling cascades, focal adhesion formation, and actin cytoskeletal organization, thus functioning as a classical α_v_ integrin agonist.

## Results

### S1-RBD of SARS-CoV2 supports cell adhesion and proliferation *via* α_v_β_3_ integrins

**Figure 1:**
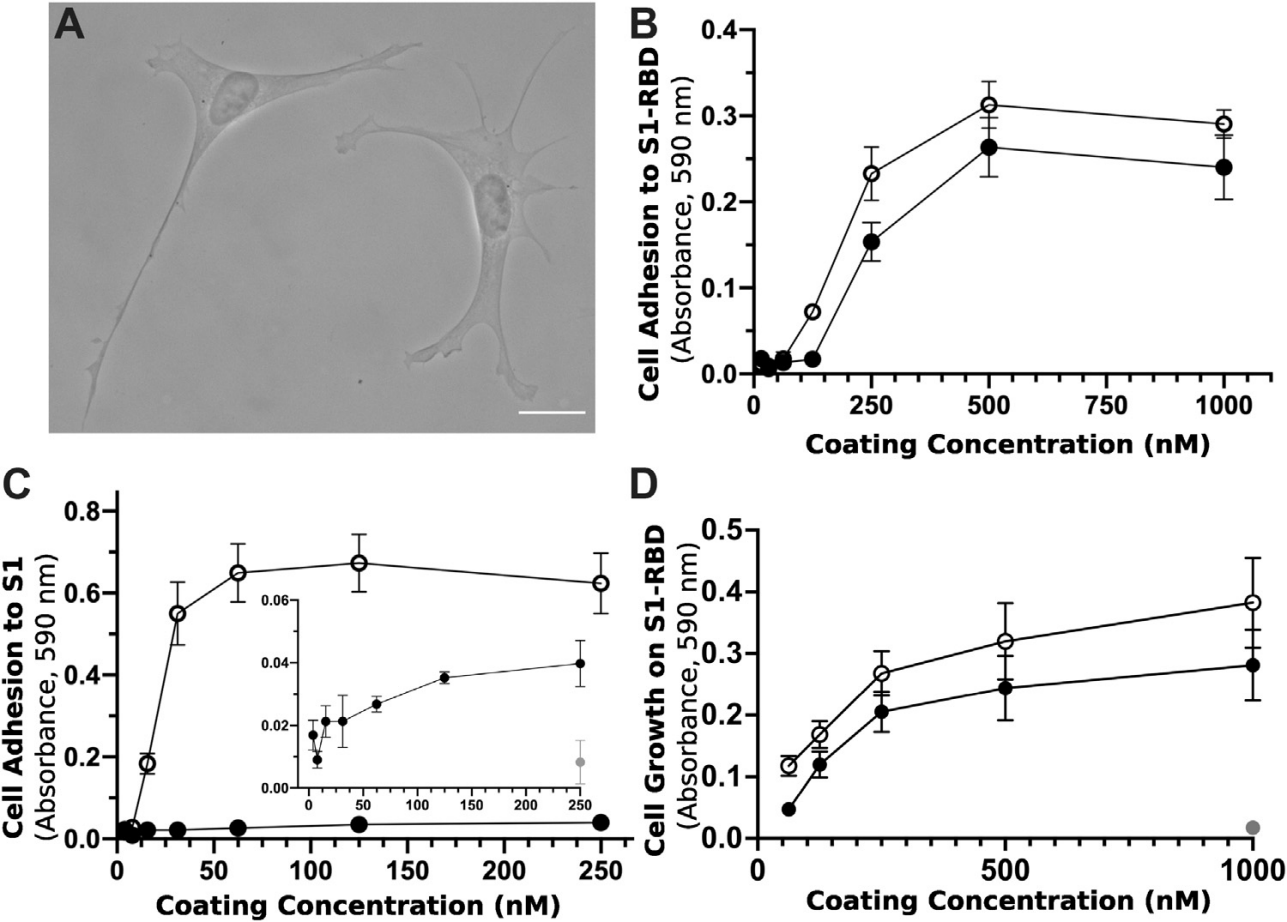
**S1-RBD supports cell adhesion and proliferation.***A*, FN-null MEFs (2.5 × 10^3^ cells/cm^2^) were seeded onto coverslips precoated with S1-RBD (500 nM) and cultured for 4 h prior to fixation and phase-contrast imaging. The scale bar represents 20 μm. *B*, FN-null MEFs (1.9 × 10^5^ cells/cm^2^) were seeded onto tissue culture plates precoated with the indicated concentration of S1-RBD (*filled circles*) or FNIII10 (*open circles*). Cells were cultured for 90 min, and relative cell number was determined by *crystal violet* staining. *C*, FN-null MEFs (1.9 × 10^5^ cells/cm^2^) were seeded onto plates precoated with HN-tagged S1 (*filled circles*) or FNIII8-13 (*open circles*) for 90 min. Inset shows cell adhesion to S1 (*filled circles*) compared with bovine serum albumin–coated wells (*gray circle*). *D*, FN-null MEFs (2.3 × 10^3^ cells/cm^2^) were seeded onto tissue culture plates precoated with the indicated concentration of S1-RBD (*filled circles*), FNIII10 (*open circles*), or GST (*gray circle*) and cultured for 4 days. Relative cell number was determined by *crystal violet* staining. Data are mean ± SEM; n ≥ 3 experiments performed in triplicate.

The integrin-binding RGD motif is contained within a variety of endogenous ECM glycoproteins (43) and is frequently expressed by microbial pathogens as a mechanism for attachment to host tissue (44). To begin to determine whether S1-RBD functionally interacts with cells, FN-null MEFs were seeded into wells coated with either S1-RBD or the RGD-containing module of fibronectin, FNIII10. At 4 h after seeding, cells adherent to S1-RBD exhibited robust adhesion and classical fibroblast morphology, characterized by extended membrane protrusions (Fig. 1*A*). Cell adhesion was dose dependent with respect to substrate coating concentration and comparable with adhesion on FNIII10 (Fig. 1*B*). Under these assay conditions, full-length S1 supported minimal cell attachment compared with the similarly sized fibronectin fragment, FNIII8-13 (Fig. 1*C*).

Ligation of integrins by RGD-containing agonists initiates cell signaling cascades that support cell proliferation (45). Thus, we next tested the ability of S1-RBD to support cell proliferation. To do so, FN-null MEFs were seeded at low density in defined, serum-free media onto tissue culture plates coated with S1-RBD, FNIII10, or the nonadhesive, protein purification tag, glutathione S-transferase (GST). After a 4-day incubation, relative cell number was quantified as a function of coating concentration. As shown in Figure 1*D*, cell number increased similarly with increasing coating density on S1-RBD- and FNIII10-coated wells. In contrast, cells seeded into GST-coated wells did not survive (Fig. 1*D*; 1 μM), indicating that cell proliferation in response to S1-RBD was specific and not due to the presence of endogenous or exogenously supplied adhesive proteins.

**Figure 2:**
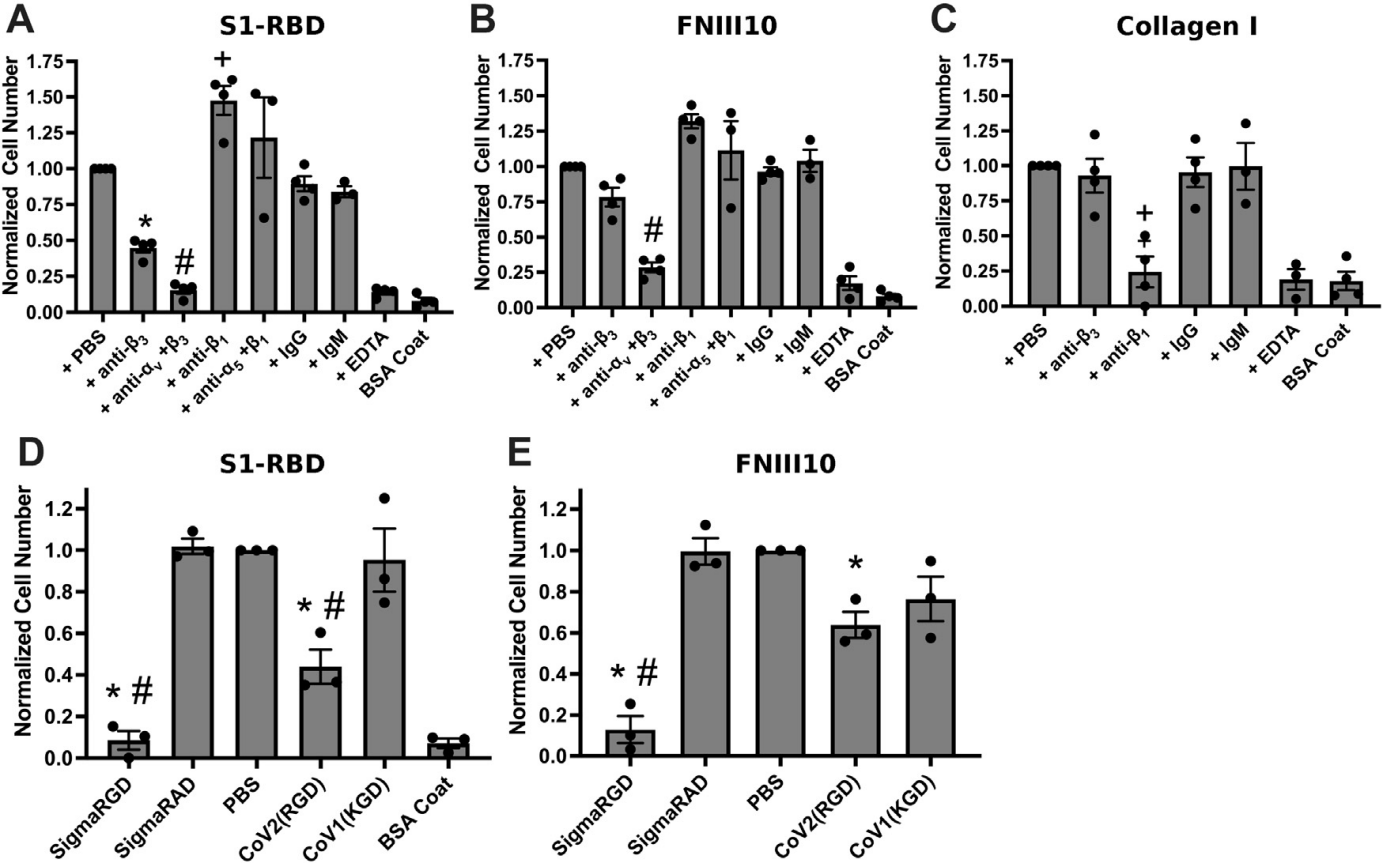
**FN-null MEF adhesion to S1-RBD is mediated by α**_**v**_**β**_**3**_**integrins and RGD.** FN-null MEFs (5 × 10^5^ cells/ml) were preincubated for 30 min with 50 μg/ml integrin-blocking antibodies (*A*–*C*) or 25 μM peptide (*D*–*E*) before seeding (9.4 × 10^4^ cells/cm^2^) onto plates precoated with 250 nM S1-RBD (*A* and *D*), FNIII10 (*B* and *E*), or type I collagen (*C*). Relative cell number was determined by *crystal violet* staining. Data are mean ± SEM, normalized to corresponding vehicle (PBS) controls; n = 3 independent experiments performed in triplicate. One-way ANOVA, Bonferroni’s post hoc test: *A*–*C* ∗*p* < 0.05 *versus* PBS, IgG; +*p* < 0.05 *versus* PBS, IgM; #*p* < 0.05 *versus* PBS, anti-α_5_+β_1_; *D*–*E* ∗*p* < 0.05 *versus* PBS; #*p* < 0.05 *versus* corresponding negative control, RAD or KGD.

Integrins are heterodimeric receptors, whose ligand specificity is determined by the combination of alpha and beta subunits (43). FN-null MEFs express α_1_, α_5_, α_v_, β_1_, and β_3_ integrin subunits (40), of which both α_5_β_1_ and α_v_β_3_ are RGD-binding integrins (43). To identify the integrin receptors mediating cell adhesion to S1-RBD, FN-null MEFs were preincubated with blocking antibodies directed against β_1_, β_3_, α_5_, or α_v_ integrin subunits. Cell adhesion to S1-RBD was inhibited partially by antibodies against β_3_-integrin subunits and inhibited completely by either a combination of α_v_- and β_3_-blocking antibodies or EDTA (Fig. 2*A*). Similar results were obtained using the α_v_β_3_-specific ligand, FNIII10 (46) (Fig. 2*B*). In contrast, cell adhesion to S1-RBD was not inhibited by either α_5_- or β_1_-blocking antibodies (Fig. 2*A*) under conditions that specifically inhibited adhesion to the β_1_-integrin ligand, collagen I (Fig. 2*C*). Rather, treatment of cells with anti-β_1_ antibodies significantly increased cell adhesion to S1-RBD (Fig. 2*A*). Finally, competitive inhibition assays were performed using short RGD-, RAD-, or KGD-containing peptides. Addition of soluble RGD peptides blocked cell adhesion to S1-RBD (Fig. 2*D*). In contrast, addition of control, RAD peptides had no effect on cell adhesion to S1-RBD (Fig. 2*D*). Furthermore, peptides derived from the RGD-containing region of SARS-CoV-2 partially inhibited cell adhesion to S1-RBD, whereas a peptide derived from the corresponding sequence of SARS-CoV-1, which contains a KGD rather than RGD motif, did not reduce adhesion to S1-RBD (Fig. 2*D*). Notably, SARS-CoV-2-derived peptides also partially inhibited cell adhesion to FNIII10 (Fig. 2*E*). Together, these data indicate that the RGD motif of S1-RBD ligates α_v_β_3_ integrins in a cation-dependent manner.

**Figure 3:**
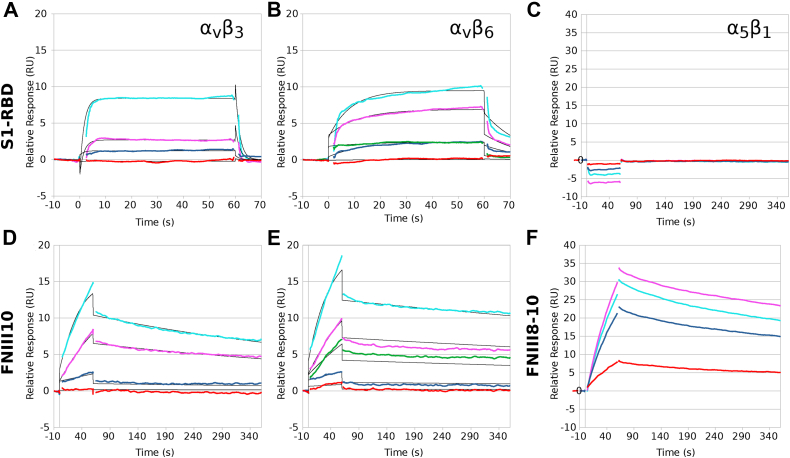
**Recombinant human integrins α**_**v**_**β**_**3**_**and α**_**v**_**β**_**6**_**, but not α**_**5**_**β**_**1**_**, bind to immobilized S1-RBD.** Representative kinetic data for α_v_β_3_ (*A* and *D*), α_v_β_6_ (*B* and *E*), or α_5_β_1_ (*C* and *F*) binding to immobilized S1-RBD (*A*–*C*), FNIII10 (*D*–*E*), or FNIII8-10 (*F*). Data are presented as representative traces (*bold colored lines*) collected from one of two (α_5_β_1_, 100–1000 nM) or three (α_v_β_3_, 5–450 nM and α_v_β_6_, 5–500 nM) experiments and corresponding 1:1 binding fits (*black lines*).

Surface plasmon resonance (SPR) was used next to study the kinetic parameters governing the binding of recombinant human integrins with immobilized S1-RBD. Representative response curves obtained from α_v_β_3_, α_v_β_6_, or α_5_β_1_ integrin binding to S1-RBD, FNIII10, or FNIII8-10 are shown in Figure 3. Experimental data were collected and globally fit using a 1:1 binding model. The fitted kinetic parameters, k_a_, k_d_, K_D_, and Rmax are shown in Table 1. The quality of each fit was evaluated by comparison with the experimentally measured Rmax and chi-squared values. Measurable binding of α_v_β_3_ integrins to S1-RBD was observed (Fig. 3*A*). However, curve-fitting parameters indicated that the goodness of fit was not sufficient to perform kinetic analysis, suggesting that the K_D_ of α_v_β_3_ integrins binding to S1-RBD is greater than 500 nM (Table 1). Kinetic modeling of data obtained for α_v_β_6_ integrin binding to S1-RBD provided a K_D_ value of 230 nM (Fig. 3*B* and Table 1). In contrast, α_5_β_1_ integrins did not bind to S1-RBD (Fig. 3*C*), in agreement with results of cell adhesion assays (Fig. 2). Measured affinities of FNIII10 binding to α_v_β_3_ (Fig. 3*D*) and α_v_β_6_ (Fig. 3*E*) integrins were 21.8 nM and 6.6 nM, respectively (Table 1), which are similar to published values (47). Association rates for the interaction of α_v_β_6_ integrins with S1-RBD and FNIII10 were similar (S1-RBD k_a_ = 8.4 × 10^4^ M^−1^s^−1^; FNIII10 k_a_ = 7.1 × 10^4^ M^−1^s^−1^). In contrast, the dissociation rate of α_v_β_6_ integrins with S1-RBD was much larger than that observed with FNIII10 (S1-RBD k_d_ = 236 × 10^−4^ s^−1^; FNIII10 k_d_ = 6.0 ×10^−4^ s^−1^). Kinetic fits were not performed for the reaction of S1-RBD and α_5_β_1_ integrins, as binding was not observed (Fig. 3*C*) even at analyte concentrations substantially exceeding the K_D_ of the interaction of FNIII8-10 with α_5_β_1_ integrins (47) and under reaction conditions in which α_5_β_1_ integrin binding to FNIII8-10 was observed (Fig. 3*F*). Together, these data indicate that S1-RBD is capable of binding directly to α_v_ integrins through low-affinity interactions.

**Table 1:** Summary of kinetic and quality control parameters determined for the interaction of α_v_ integrins with immobilized S1-RBD and FNIII10

Ligand	Analyte	k_a_ × 10^4^	k_d_ × 10^−4^	K_D_ (nM)	Rmax fit (RU)	Rmax measured (RU)	Chi^2^
S1-RBD	α_v_β_3_	-	-	>500	-	-	-
S1-RBD	α_v_β_6_	8.4 ± 1.8	236 ± 206	230 ± 180	6.9 ± 3.5	11.7 ± 0.7	0.1 ± 0.3
FNIII10	α_v_β_3_	7.1 ± 1.4	15.0 ± 1.8	21.8 ± 1.6	12.8 ± 0.5	12.6 ± 0.4	0.06 ± 0.01
FNIII10	α_v_β_6_	25.0 ± 18.8	6.0 ± 0.7	6.6 ± 3.1	16.6 ± 2.5	19.0 ± 2.3	0.16 ± 0.05

Data are presented as mean ± SEM for at least three independent experiments per integrin. Double-referenced experiments were performed simultaneously for S1-RBD and FNIII10 ligands in parallel flow cells.

### S1-RBD initiates focal adhesion formation and actin organization

**Figure 4:**
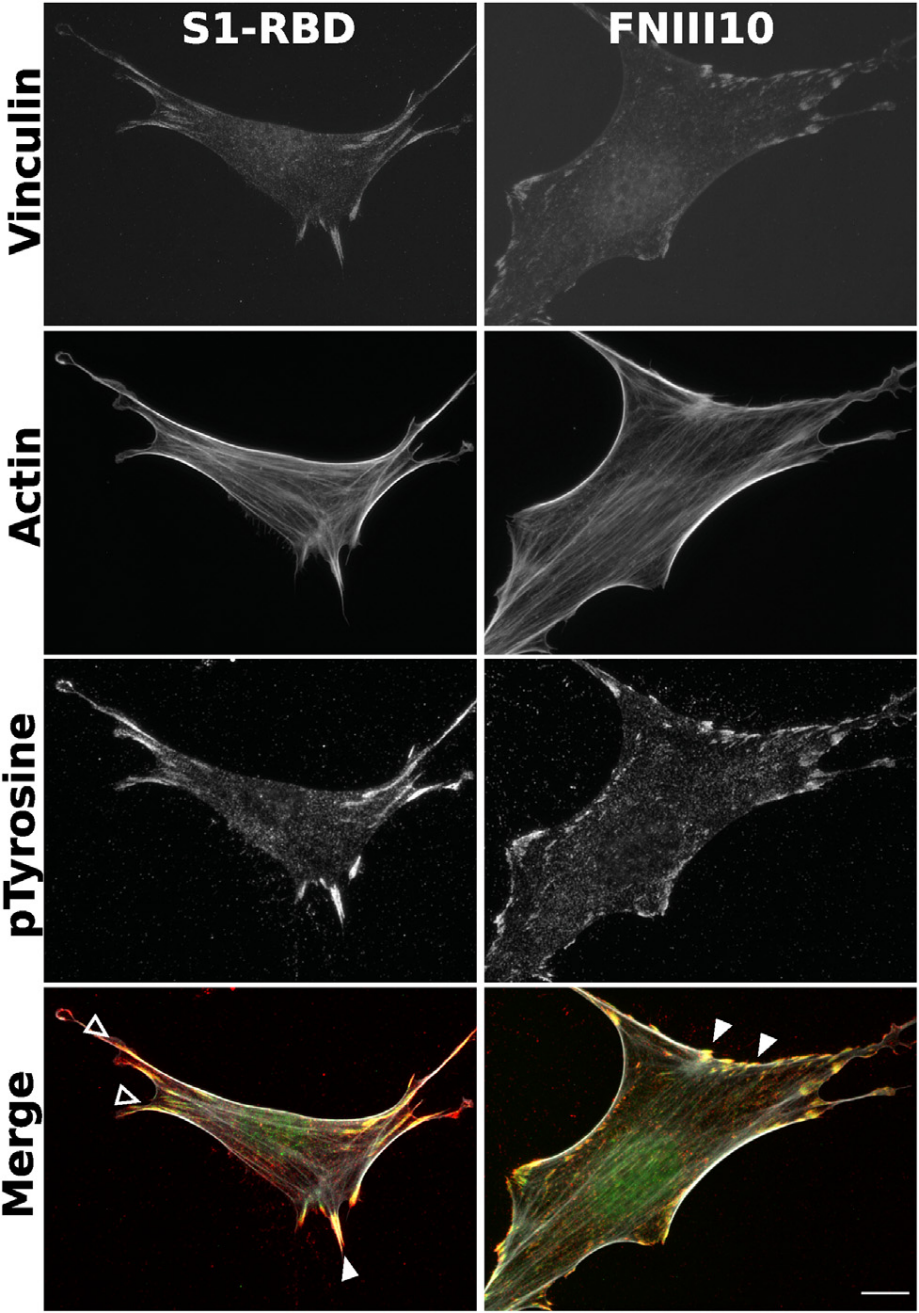
**S1-RBD engagement initiates focal adhesion formation and actin organization.** FN-null MEFs (2.5 × 10^3^ cells/cm^2^) were seeded on coverslips coated with 500 nM S1-RBD (*left*) or FNIII10 (*right*). Cells were incubated for 4 h prior to fixation and immunofluorescent staining for vinculin (*green*), actin (TRITC-phalloidin, *white*), or phosphotyrosine (4G10, *red*). *Arrowheads* represent colocalization of vinculin and phosphotyrosine within focal adhesions (closed) and engagement with the actin cytoskeleton (open). Representative images shown from one of four independent experiments. The scale bar represents 10 μm.

Integrin ligation by endogenous ECM ligands triggers adhesion signaling cascades in which intracellular mediators are recruited to sites of integrin activation (45, 48). These protein complexes, known as focal adhesions, serve as central signaling hubs and functionally couple ECM-engaged integrins to the actin cytoskeleton (49). Notably, manipulation of focal adhesion signaling has been identified across a diverse spectrum of microbial pathogens, with the potential to influence multiple stages of cellular pathophysiology, including cell-surface attachment, invasion, and cell death (44). To determine whether engagement of α_v_β_3_ integrins by S1-RBD supports focal adhesion formation and downstream signaling, FN-null MEFs adherent to S1-RBD were stained with the actin-binding protein phalloidin, together with antibodies against the focal adhesion adaptor vinculin, and a pan-specific phosphotyrosine antibody (50). Cells adherent to S1-RBD- or FNIII10-coated substrates were well spread and exhibited classical features of focal adhesions, including colocalized vinculin and phosphotyrosine staining, as well as actin stress fiber formation (Fig. 4). S1-RBD-adherent cells typically exhibited fewer, but larger, focal contacts than FNIII10-adherent cells.

**Figure 5:**
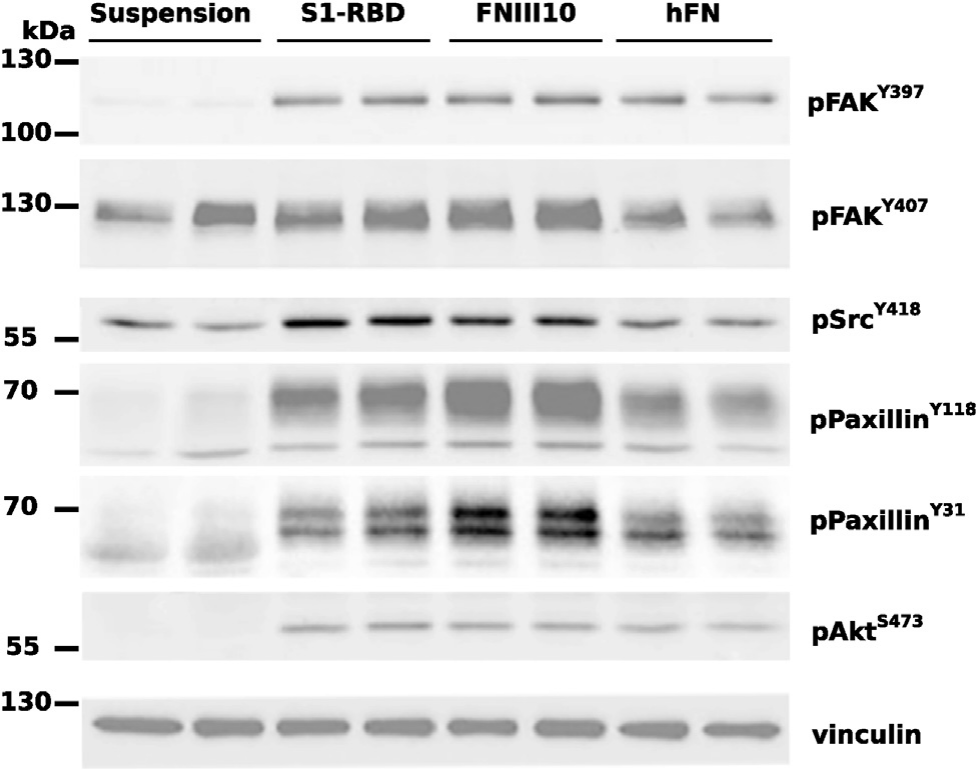
**Cell engagement with S1-RBD stimulates intracellular signaling.** FN-null MEFs were either suspended in media or seeded at 3.5 × 10^5^ cells/cm^2^ on wells precoated with 500 nM of S1-RBD or FNIII10, or 10 μg/ml of human plasma fibronectin (hFN) for 1 h. Cell lysates were analyzed by immunoblotting with the indicated phosphospecific antibodies or vinculin, as a loading control. Molecular mass markers are shown on the *left*.

To identify proteins specifically phosphorylated by S1-RBD ligation, immunoblot analysis of whole-cell lysates was performed. Similar patterns of protein tyrosine phosphorylation were observed when lysates from attached S1-RBD- or FNIII10-adherent cells were probed with a pan-specific phosphotyrosine antibody (not shown). As such, immunoblots were next probed with phosphospecific antibodies against key components of adhesion signaling pathways (Fig. 5). These components included the early, adhesion-dependent autophosphorylation of focal adhesion kinase (FAK) at Y^397^ (51), which in turn enables recruitment and phosphorylation of Src at Y^418^ (52). Both FAK-Y^397^ and Src-Y^418^ were phosphorylated in response to S1-RBD ligation (Fig. 5). Moreover, the extent of FAK and Src phosphorylation was similar to that observed in either FNIII-10- or fibronectin-adherent cells (Fig. 5). FAK may be phosphorylated at additional tyrosine residues including Y^407^ (53), which was phosphorylated to a similar extent in both suspended and adherent cells (Fig. 5). S1-RBD triggered tyrosine phosphorylation of paxillin (Fig. 5), a central adaptor protein whose SH2 domains require phosphorylation at residues Y^118^ and Y^31^ for activation and cytoskeletal remodeling (54, 55). In addition, S1-RBD induced Akt phosphorylation at residue S^473^ to a similar extent as FNIII10- and fibronectin-adherent cells, implicating engagement of the prosurvival PI3K/Akt signaling axis (56, 57) and consistent with results demonstrating that S1-RBD ligation supports cell proliferation (Fig. 1*D*). Together, these data indicate that S1-RBD can trigger multiple aspects of adhesion-based signaling, including localization of vinculin to focal adhesions, phosphorylation of early adhesion signals FAK and Src, as well as activation of downstream adhesive effectors, including paxillin and Akt.

**Figure 6:**
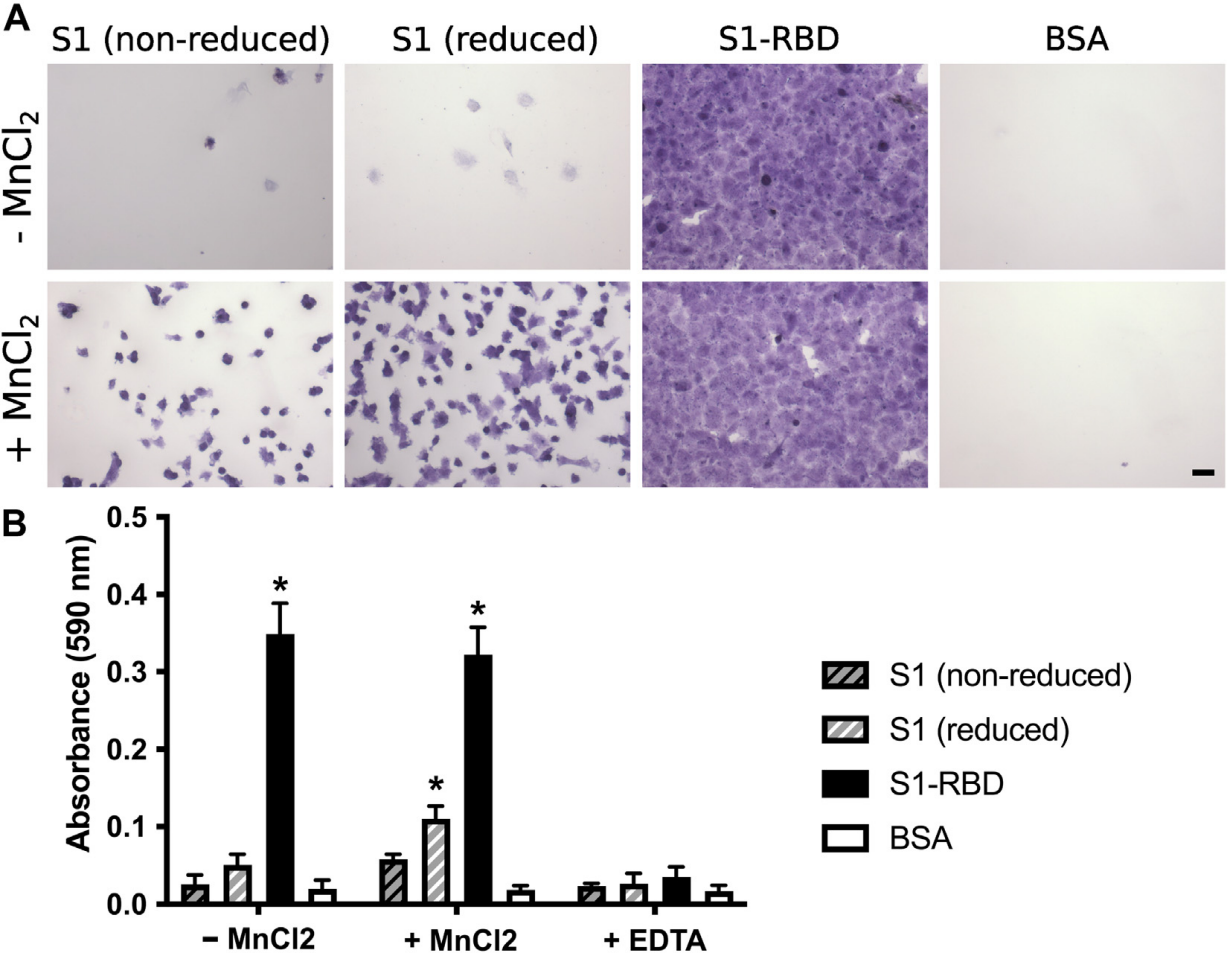
**S1 contains a cryptic, Mn**^**2+**^**-sensitive adhesive epitope.***A*, FN-null MEFs (9.4 × 10^4^ cells/cm^2^) were seeded on wells precoated with 250 nM reduced or nonreduced S1 protein, S1-RBD, or 1% bovine serum albumin (BSA). Cells were seeded for 1 h in the absence or presence of 1 mM MnCl_2_ or 10 mM EDTA. *A*, representative images of *crystal violet*–stained cells. The scale bar represents 100 μm. *B*, relative cell number presented as mean absorbance ± SEM, n = 3 independent experiments performed in triplicate. ∗*p* < 0.05 *versus* corresponding BSA control by two-way ANOVA with Bonferroni’s post hoc test.

The data presented thus far indicate that the RGD sequence within S1-RBD is a functional, integrin-binding ligand that can mimic classical features and functions of native ECM ligands. In contrast, cells attached poorly to a larger fragment of S1 (Fig. 1*C*), which contains both the N-terminal domain and furin cleavage site in addition to RBD. Cryptic adhesive epitopes are a feature common to many native ECM proteins, including fibronectin (58) and thrombospondin (59). Thus, studies were conducted to explore conditions that might promote cell interactions with the larger S1 fragment. First, S1 was chemically reduced to alter its conformation. Cell adhesion to substrates precoated with either reduced or nonreduced S1 was then determined in the absence and presence of MnCl_2_, a potent activator of α_v_β_3_ integrins (60). In the absence of MnCl_2_, few cells attached to S1, either in the nonreduced or reduced form (Fig. 6*A*, S1 - MnCl_2_). In contrast, in the presence of MnCl_2_, cells were visibly attached and well spread on substrates coated with either nonreduced or reduced S1, compared with bovine serum albumin (BSA)-coated wells (Fig. 6*A*, S1 + MnCl_2_). Cells attached and spread similarly on S1-RBD-coated substrates in the absence and presence of MnCl_2_ (Fig. 6*A*, S1-RBD ± MnCl_2_) indicating that integrin interaction with RBD did not require the high-affinity state.

Cell attachment to S1 was quantified using adhesion assays. In the presence of MnCl_2_, cell attachment to wells coated with reduced S1 was statistically increased *versus* BSA-coated wells (Fig. 6*B*; reduced S1 + MnCl_2_ *versus* BSA + MnCl_2_). Moreover, adhesion to reduced S1 was sensitive to the metal ion chelator, EDTA (Fig. 6*B*, reduced S1 ± EDTA). To determine whether increased cell adhesion to reduced S1 was due to changes in S1 protein conformation or increased substrate coating efficiency, ELISAs were performed on substrate-coated wells using anti-His antibodies. The relative coating density of reduced S1 was approximately double that of nonreduced S1 and S1-RBD (mean absorbances ± SD: reduced S1 = 1.56 ± 0.04; nonreduced S1 = 0.76 ± 0.01; S1-RBD = 0.95 ± 0.08). As such, the increase in cell adhesion observed with reduced S1 protein was likely due to an increase in coating density of the reduced S1 protein. There was no significant difference in protein density of wells coated with nonreduced S1 *versus* RBD. Together, these data indicate that, within the larger S1 fragment, the adhesive capacity of RBD is detectable but substantially reduced *versus* the RBD fragment.

### S1-RBD exhibits cation- and RGD-dependent binding to primary human small airway epithelial cell monolayers

**Figure 7:**
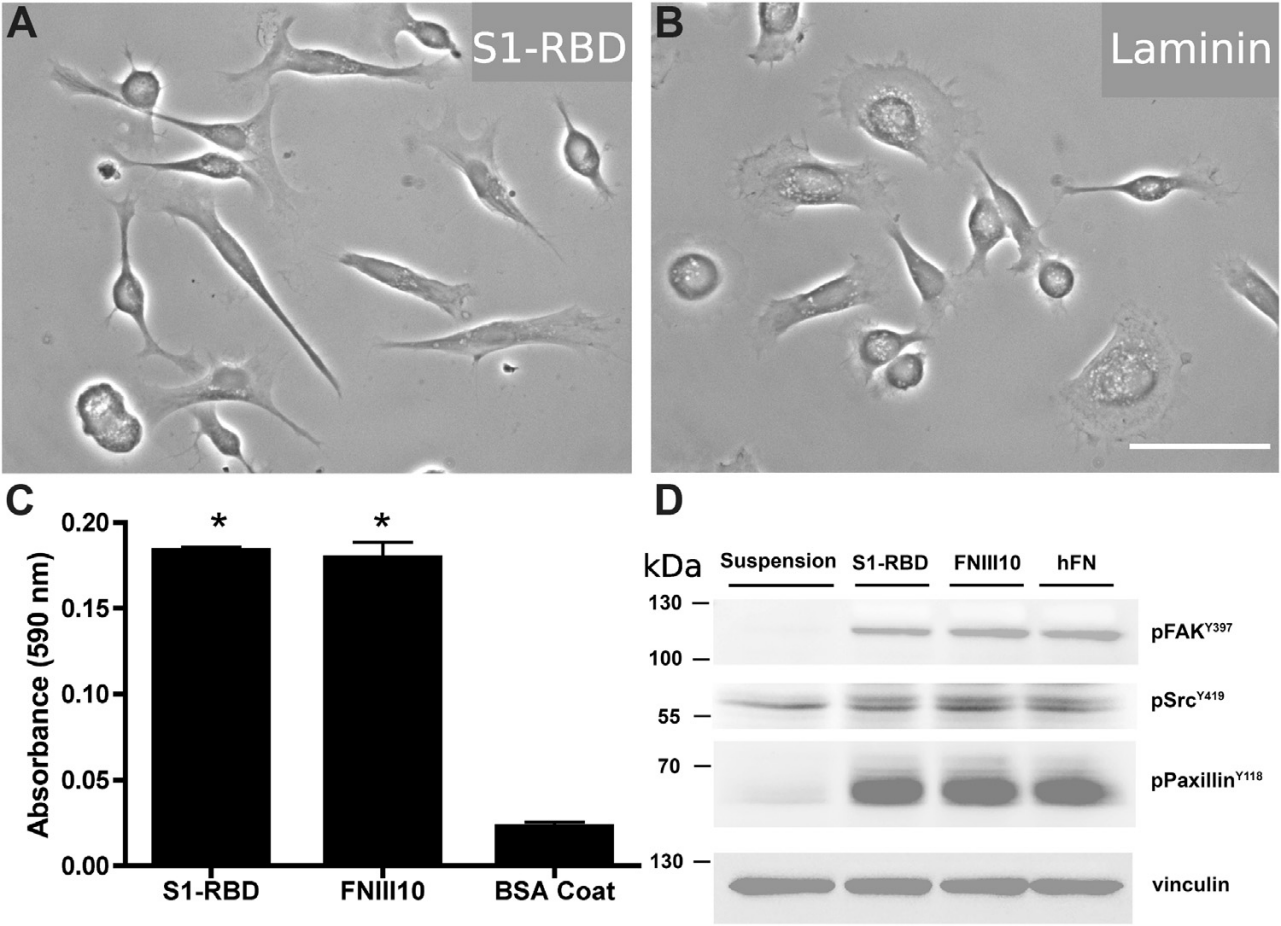
**S1-RBD supports cell adhesion and phosphotyrosine signaling in human small airway epithelial cells.** hSAECs (6.7 × 10^4^ cells/cm^2^) were seeded in the presence of 1 mM MnCl_2_ on wells precoated with S1-RBD, laminin, FNIII10, or 1% bovine serum albumin (BSA). Representative images of SAECs adherent of S1-RBD (*A*) or laminin (*B*) are shown. The scale bar represents 50 μm. *C*, cell adhesion after 2 h was determined by *crystal violet* staining. Data are presented as mean absorbance ± SEM. ∗*p* < 0.05 *versus* BSA by one-way ANOVA with Bonferroni’s posttest. *D*, cell lysates were obtained after 4 h of adhesion and analyzed by immunoblotting with the indicated phosphospecific antibodies. Vinculin was used as a loading control. Molecular mass markers are shown on the *left*. Control cells were maintained suspended in medium before lysing. SAEC, small airway epithelial cell.

We next sought to determine whether S1-RBD can mediate adhesion of human primary small airway epithelial cells (hSAECs). These primary cells are derived from the distal lung and are susceptible to SARS-CoV-2 infection (61, 62). hSAECs seeded in the presence of 1 mM MnCl_2_ attached and spread on S1-RBD-coated wells to a similar extent as that observed with laminin-coated wells (Fig. 7, *A* and *B*). hSAEC adhesion to S1-RBD was significantly increased compared with BSA-coated wells and was similar to that observed with wells coated with the αvβ3-ligand, FNIII10 (Fig. 7*C*). Furthermore, hSAECs adhesion to S1-RBD stimulated tyrosine phosphorylation of FAK, paxillin, and Src to a similar extent as FNIII10 and fibronectin (Fig. 7*D*). Thus, S1-RBD stimulates adhesion-mediated intracellular signaling pathways in human lung epithelial cells.

**Figure 8:**
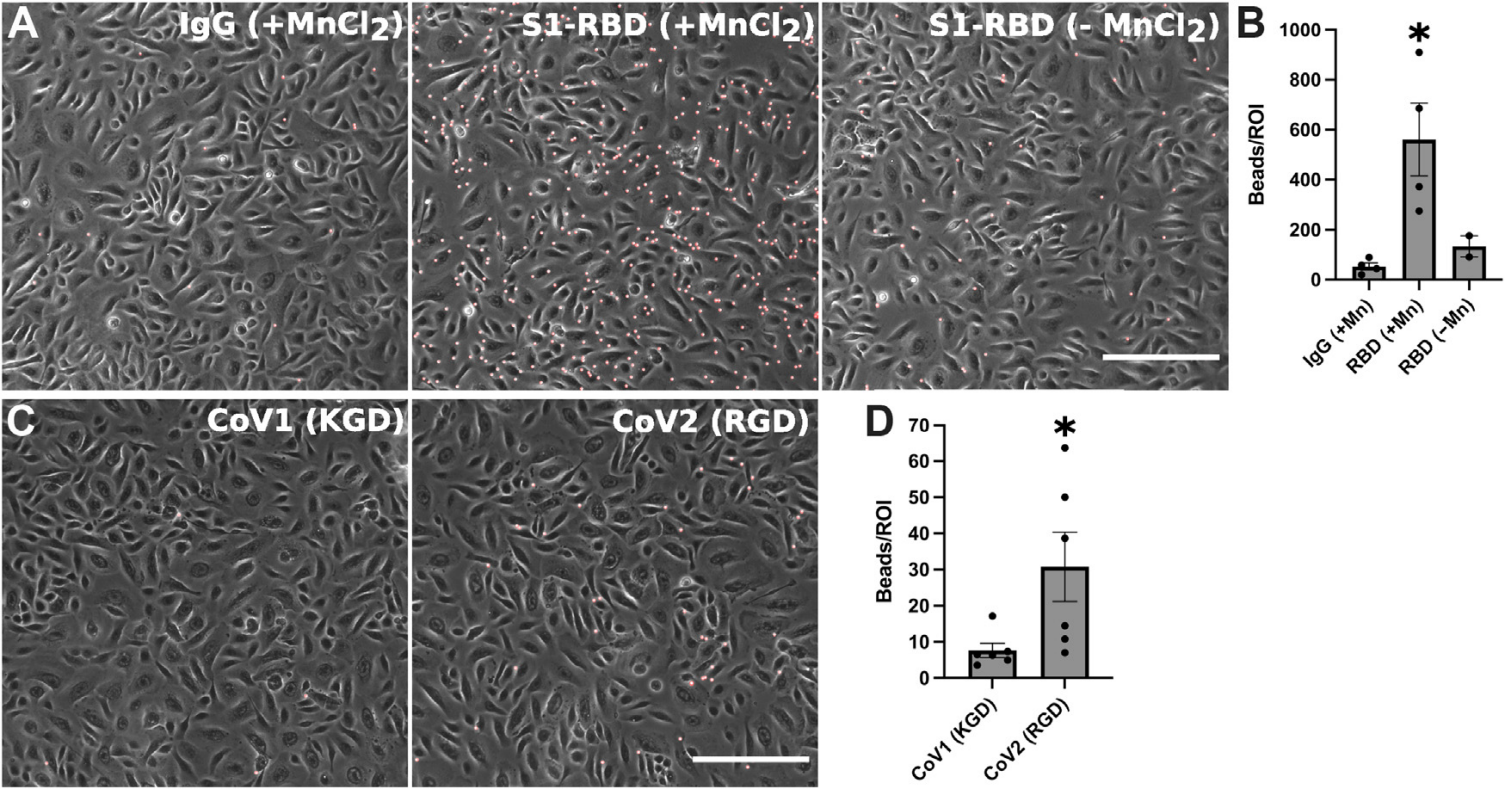
**S1-RBD-bound beads bind to SAEC monolayers in a cation- and RGD-dependent manner.***A*, laminin-adherent SAEC monolayers were treated with (*A* and *B*) IgG- or Fc-RBD-immobilized fluorescent microbeads in the presence or absence of 1 mM MnCl_2_ or (*C* and *D*) biotinylated CoV-2(RGD) or CoV-1(KGD) peptide-immobilized fluorescent microbeads in the presence of 1 mM MnCl_2_. Cells were incubated for 2 h at 4 °C. (*A* and *C*) representative phase images are shown; protein-immobilized beads are *red*. The scale bar represents 200 μm. (*B* and *D*) bead binding to SAEC monolayers was quantified as the mean for three independent regions of interest (ROIs) per well. Data are mean number of beads bound per 0.6 mm^2^ ROI ± SEM. In (*B*), n = 4 (+Mn) or n = 2 (−Mn) replicates per condition on two independent experimental days. ∗*p* < 0.05 *versus* IgG by one-way ANOVA with Bonferroni’s post hoc test. In (*D*), n = 6 replicates per condition on three independent experimental days. ∗*p* < 0.05 *versus* KGD by two-tailed *t* test. SAEC, small airway epithelial cell.

Thus far, the ability of S1-RBD to bind to integrins has been analyzed by presenting immobilized S1 fragments to nonadherent cells. To evaluate S1-RBD binding to cells that are already adherent and spread on native ECM substrates, assays were performed using either Fc-tagged RBD fragments immobilized on fluorescent protein G–coupled microbeads or biotin-tagged CoV 20-mer peptides immobilized on streptavidin-coupled microbeads. To begin, hSAEC monolayers were incubated with RBD- or IgG-immobilized beads. Following a 2-h incubation, limited binding of IgG-coupled beads to hSAEC monolayers was observed (Fig. 8*A*; IgG + MnCl2). In contrast, RBD-immobilized beads attached extensively to hSAECs in the presence but not the absence of MnCl_2_ (Fig. 8*A*). Quantification of the number of beads bound per imaging field indicated a significant increase in the number of RBD-beads bound *versus* IgG controls (Fig. 8*B*), demonstrating cation-dependent binding of S1-RBD to epithelial cell surfaces.

The role of the RGD motif in mediating RBD binding to epithelial cell surfaces was next assessed using CoV-derived peptides. A 20-mer, CoV-2 peptide encompassing the RGD motif was synthesized, and binding to epithelial cell surfaces was compared with the corresponding peptide of CoV-1, which contains a KGD sequence in place of RGD. The CoV-1 sequence was chosen expressly as a control for CoV-2 binding, as KGD is also an integrin-binding motif but with specificity for platelet αIIbβ3 integrins (63), which are not expressed by primary epithelial cells (64). Peptide-bound microbeads were incubated with laminin-adherent SAECs for 2 h, and unbound beads were removed by washing. As shown in Figure 8*C*, microbeads coated with RGD-containing CoV-2 peptides readily attached to epithelial cell surfaces, whereas beads coated with KGD-containing CoV-1 peptides did not. Quantification of the number of beads bound per imaging field indicated a significant increase in the number of CoV-2- *versus* CoV-1-beads bound per region of interest (Fig. 8*D*). The CoV-1- and CoV-2-derived peptides utilized in this study differ only in their integrin-binding sequences (IRGDE *versus* VKGDD for CoV-2 and CoV-1, respectively), thus providing additional evidence that mutation of the KGD sequence of CoV-1 to RGD conferred the RBD of CoV-2 with the capacity to interact with epithelial cell integrins.

## Discussion

The identification of a conserved RGD motif within the SARS-CoV-2 spike protein generated substantial scientific interest (21, 33), and converging lines of experimental evidence suggest that integrin inhibition may be protective against SARS-CoV-2 binding and infection (34, 36, 37, 38, 65). To the best of our knowledge, the data presented in the present study are the first to demonstrate that the receptor-binding domain of SARS-CoV-2 spike protein functions as a classical integrin receptor agonist. S1-RBD supported cell adhesion (Fig. 1, *A* and *B*) and proliferation (Fig. 1*D*) to comparable extents as the RGD-containing fragment of the native ECM molecule fibronectin (FNIII10). This interaction was competitively inhibited by both α_v_ integrin-blocking antibodies and RGD peptides (Fig. 2, *A* and *D*) and was also observed in an SPR model of direct S1-RBD-integrin binding (Fig. 3). Cells adherent to S1-RBD formed focal adhesions (Fig. 4), and key adhesion signaling mediators FAK, Src, Paxillin, and Akt were phosphorylated (Fig. 5). The present studies were conducted primarily in mouse embryonic fibroblasts, which do not express detectable levels of ACE2, and thus these results are unlikely to be complicated by potential interactions of S1-RBD with ACE2. As well, S1-RBD supported both cell attachment and adhesion-based signaling in primary human small airway epithelial cells (Fig. 7), while RBD-bound microbeads attached readily to SAEC monolayers (Fig. 8). Together, these results demonstrate that SARS-CoV-2 spike protein contains a functional adhesive epitope within the RBD that mediates α_v_ integrin engagement *via* its RGD motif.

Of reports investigating integrin-spike interactions, α_5_β_1_ has been proposed as a receptor of interest, in part due to its functional association with ACE2 (66, 67), the ability of β1-selective integrin antagonists to reduce SARS-Co-V2 invasion (34, 37, 38), and observed α_5_β_1_ integrin-S1-RBD interactions by ELISA (34) and SPR (68) assays. Some reports have also implicated α_v_β_3_ integrins in viral entry (36, 69), while others found no effects of integrin antagonists on viral invasion (33). In the present study, we compared effects of β_1_- and β_3_-integrin-blocking antibodies on cell attachment to RBD using a well-characterized fibroblast cell line that expresses both functional α_5_β_1_ and α_v_β_3_ integrin receptors (40, 41). Notably, FN-null MEFs do not produce fibronectin, are cultured in the absence of serum, and do not deposit other endogenous matrix molecules, including RGD-containing thrombospondin and the α_1/2_β_1_ integrin ligand collagen, into their ECM (42). FN-null MEFs adhered to S1-RBD exclusively *via* α_v_β_3_ integrins, with no contribution from α_5_β_1_ integrins (Fig. 2, *A*–*C*). This result was confirmed using recombinant integrins and SPR, which further indicated that the affinity of the epithelial integrin α_v_β_6_ for S1-RBD was substantially higher than that of α_v_β_3_ (Fig. 3 and Table 1). In contrast to previous reports (68), we found no detectable interaction between S1-RBD and α_5_β_1_ integrin using SPR. This intriguing finding may be due to differences in the SPR conditions, which in the present study, included the use of the nonionic detergent octyl glucoside (70) and MnCl_2_ (60, 71) to support functional activation of integrins within a purified protein system. While we did not test the integrin specificity of the larger S1 fragment in the present study, recent work by Park and colleagues (65) showed that an Fc-tagged S1 fragment could support α_v_-, α_4_-, or β_1_-mediated adhesion depending on cell type–specific integrin expression. Thus, the possibility remains that, like fibronectin (46, 47), synergistic sequences or conformational flexibility within the larger S1 domain may confer additional, dynamic integrin selectivity. The specificity and selectivity of spike and spike fragments may further be sensitive to modulators of integrin signaling, such as heparin sulfate proteoglycan coreceptors (72) or cell-surface proteases (73), both of which have been identified as factors regulating engagement of SARS-CoV-2 virions with host target cells (31, 33, 74, 75).

In contrast to the robust adhesive response observed on S1-RBD, cells seeded onto the larger S1 fragment of SARS-CoV-2 spike protein attached only weakly and exhibited limited spreading (Figs. 1*C* and 7B). Cell adhesion to S1 was partially rescued by chemical reduction of S1, which increased coating efficiency, and pretreatment of cells with Mn^2+^. One possible interpretation of these data is that the adhesive epitope contained within S1 is cryptic and thus only available to integrins under appropriate physical and chemical conditions. This hypothesis is further supported by molecular dynamics simulations suggesting that, in the absence of other interactions, the RGD site is unable to adopt the geometry necessary for high-affinity integrin ligation (76). Matricryptic epitopes have been identified in a number of native ECM proteins, including thrombospondin (59), which contains a cryptic RGD sequence whose exposure is regulated by cell-surface protein disulfide isomerases (77). Likewise, exposure of a self-association epitope in fibronectin (58) can be exposed by cell-derived mechanical force (78) and proteolytic fragmentation (58). Thus, the specific conformational requirements and activation steps enabling functional engagement of integrin receptors with SARS-CoV-2 spike in the context of established mechanisms of viral attachment and invasion represents an open question of substantial importance.

The intact, trimeric SARS-CoV-2 spike undergoes multiple conformational changes and molecular interactions during the viral invasion process (79), including conformational flexibility of the RBD domain (80, 81), as well as activation by cell-surface proteases TMPRSS2 (31) and Cathepsin L (33, 75). Separation of the RBD from the prefusion spike trimer during proteolytic activation may therefore be a critical activation step prior to integrin binding. Furthermore, the well-characterized, cell-surface spike receptor ACE2 (31, 33) and the more recently identified coreceptor heparin sulfate proteoglycans (74, 82) can associate laterally with integrins on human cell surfaces (66, 67, 83, 84). Thus, integrins and associated intracellular signaling partners are emerging as putative components of a larger molecular complex that is targeted during SARS-CoV-2 infection. Differences in baseline integrin expression and activation state between cell lines are also a likely contributing factor in conflicting reports on the integrin dependence and selectivity of SARS-CoV-2 infection (33, 34, 36, 37, 38, 69). Future elucidation of the conformational requirements and activation steps enabling functional engagement of integrin receptors with SARS-CoV-2 spike in the context of established mechanisms of viral attachment and invasion represents an open question of substantial importance.

The demonstration of an α_v_-specific integrin agonist functionality contained within S1-RBD protein opens multiple avenues that will be critical in expanding scientific understanding of SARS-CoV-2 and therapeutic options for a global population affected by the COVID-19 pandemic. The most immediate among these, the identification of anti-integrin therapeutics that are US Food and Drug Administration approved or in preclinical trials with potential efficacy against SARS-CoV-2 infection, is already underway (85). Integrins have been implicated in the pathophysiology of numerous respiratory viruses, including human cytomegalovirus (86), hantaviruses (87), and influenza (88), as either primary receptors or as major mediators of host response and disease severity, with the specific contributions of integrins in the context of COVID-19 disease yet to be elucidated (21, 89). Meanwhile, numerous questions remain unanswered regarding mechanisms underlying the differential susceptibility of vulnerable populations to severe manifestations of COVID-19 (3), as well as potential differences in infectivity, transmissibility, disease severity, and immune evasion associated with novel variants (https://covariants.org/) (90). Interrogating these open challenges in the context of integrin-spike interactions, including factors determining integrin selectivity and specificity, is a promising and yet unexplored avenue. For example, ACE2 cell surface expression levels alone do not sufficiently predict tissue susceptibility or disease severity (91). Thus, a combinatorial expression profile of ACE2, alongside α_v_ integrin surface expression, may better predict cell tropism of SARS-CoV-2. Alternatively, fibronectin–integrin interactions play a key role in maintaining endothelial barrier function during sepsis (15, 16, 92, 93, 94, 95), which may be disrupted by competition from spike protein fragments during SARS-CoV-2-driven inflammation, a hypothesis that is supported by recent work in vascular endothelial cells (35). Variations in integrin expression and activation state are likewise associated with some of the key risk factors for severe complications of SARS-CoV-2 infection (3), including diabetes (96, 97), hypertension (98, 99, 100), and differing inflammatory responses (88, 101). As the global COVID-19 pandemic approaches a new, endemic stage, targeting the emerging spike-integrin signaling axis has the potential to become an essential tool in preventing or mitigating the most severe effects of the disease, particularly for vulnerable patients who are not fully protected by current preventative and therapeutic regimens.

## Conclusions

The SARS-CoV-2 spike protein contains a novel RGD motif within its receptor-binding domain (S1-RBD). We demonstrate that S1-RBD is a functional integrin agonist with selectivity for α_v_ integrins, specifically α_v_β_3_ and α_v_β_6_. In contrast, we found no evidence of S1-RBD engagement with α_5_β_1_ integrins in either cellular adhesion or SPR systems. S1-RBD-mediated cellular adhesion supported cell spreading and cytoskeletal engagement, focal adhesion formation, and stimulation of key intracellular signaling pathways associated with cytoskeletal organization and cell proliferation. Together, these data point to a functional role for α_v_ integrins during attachment and invasion of SARS-CoV-2 and provide insight into critical open questions regarding COVID-19 pathophysiology, including mechanisms underlying variable disease severity, intersecting risk factors, and post-acute viral sequelae.

## Experimental procedures

### Reagents

Fibronectin was purified from outdated human plasma (American Red Cross) using gelatin-Sepharose (GE Life Sciences, now Cytiva) affinity chromatography (102). Type I collagen (rat tail) was purchased from Corning (354236). Unless otherwise indicated, chemicals were obtained from J.T. Baker or Sigma-Aldrich. GST-tagged FNIII10 and HN-tagged FNIII10-13 were produced and purified from *Escherichia coli* as described (46, 103). His-tagged S1 and S1-RBD of SARS-CoV-2 were purchased from Sino Biological (40591-V08H) and R&D Systems (10523-CV), respectively. Fc-tagged S1-RBD was from R&D Systems (10565-CV). Where indicated, S1 was reduced by successive 1-h treatments with 10 mM DTT and 30 mM N-ethyl maleimide (NEM) at 37 °C. Both reduced and nonreduced S1 were dialyzed into PBS prior to use. Integrin-blocking antibodies anti-α_5_ (clone 5H10-27), anti-α_V_ (clone H9.2B8), anti-β_1_ (clone Ha2/5), and anti-β_3_ (clone 2C9.G2) and isotype controls were purchased from BD Biosciences. Antibodies for immunofluorescent staining were as follows: vinculin (clone VIN-11-5, Sigma or clone 42H89L44, Invitrogen); phosphotyrosine (clone 4G10, Sigma or PY20, BD Biosciences); phospho-FAK pY407 (polyclonal, Invitrogen #44650G); phospho-FAK pY397 (polyclonal, Biosource #44-624G); phospho-Src pY418 (polyclonal, Biosource #44-660); phospho-Paxillin pY118 (polyclonal, Invitrogen #44-722G); phospho-Paxillin pY31 (polyclonal, Invitrogen #44-720G); phospho-Akt pS473 (polyclonal, Cell Signaling #9271); TRITC-labeled phalloidin (Millipore, #90228). Alexa Fluor–conjugated secondary antibodies were from Molecular Probes. RGD-containing peptides derived from SARS-CoV-2 (ADSFVIRGDEVRQIAPGQTG) and KGD-containing peptides derived from SARS-CoV (ADSFVVKGDDVRQIAPGQTG) were produced with and without an N-terminal biotin-Ahx tag by Genscript. Integrin-blocking (GRGDSP, #SCP0157) and negative control (GRADSP, #SCP0156) peptides were purchased from Sigma. Recombinant human integrins αvβ3 (3050-AV), αvβ6 (3817-AV), and α5β1 (3230-A5) were from R&D Systems. Protein G–coated pink (PGFP-5058-5, 5.0–5.9 μm diameter) and streptavidin-coated Nile red (SVFP-6056-5, 5.0–7.9 μm diameter) fluorescent particles were purchased from Spherotech, Inc.

### Cell culture

FN-null MEFs, derived previously from homozygous fibronectin knockout mouse embryos (40), were cultured under serum- and fibronectin-free conditions on collagen I–coated tissue culture flasks using a 1:1 mixture of Aim V (Invitrogen) and Corning SF Medium (Corning), as described (40). FN-null MEFs do not express vitronectin or laminin (39, 40) and in the absence of supplemental fibronectin are unable to assemble ECM fibrils of collagen I (104), thrombospondin (42), or fibrinogen (105). Adult human small airway epithelial cells (SAECs) were purchased from Lonza (CC-2547) and used between passages 6 and 8. SAECs were cultured in serum-free Small Airway Epithelial Growth Medium (Lonza CC-3118), according to manufacturer’s instructions. Cells were passaged at 70 to 80% confluence using ReagentPack subculture reagents (Lonza CC-5034). Neither FN-null MEFs nor SAECs expressed detectable levels of ACE2 protein by immunoblot analysis (data not shown).

### Cell adhesion and proliferation assays

Cell adhesion assays were performed as described (41). Briefly, 96-well tissue culture plates were coated with S1-RBD (10–1000 nM), FNIII10 (10–1000 nM), GST (1000 nM), or S1 (7.8–250 nM) for 1 h at 37 °C. Relative protein coating concentrations were quantified by enzyme-linked immunoabsorbent assays (ELISA) using anti-His antibodies, as described (106). Cells were seeded on protein-coated wells (9.4 × 10^4^ cells/cm^2^) in either AimV/SF medium (FN-null MEFs) or Small Airway Epithelial Basal Medium (CC-3119; Lonza) in the absence or presence of EDTA (10 mM), DTT (1 mM), or MnCl_2_ (1 mM) as indicated; MnCl_2_ was added 1 h after seeding. For integrin blocking studies, FN-null MEFs were preincubated with anti-integrin antibodies (50 μg/ml) or 25 μM peptide for 1 h prior to seeding. Integrin-blocking studies were performed using subsaturating protein coating concentrations to reduce the amount of antibody or peptide required to inhibit adhesion. Cells were then seeded into wells and incubated at 37 °C and either 8% (FN-null MEFs) or 5% (SAECs) CO_2_ for up to 2 h. Wells were washed with PBS to remove nonadherent cells, fixed with 1% paraformaldehyde, and stained with 0.5% crystal violet. The absorbance of crystal violet solubilized in 1% SDS was measured at 590 nm. Proliferation assays were performed by seeding FN-null MEFs (2.5 × 10^3^ cells/cm^2^) on protein-coated 48-well plates. Cells were cultured for 4 days at 37 °C, 8% CO_2_ and then fixed and stained with crystal violet (41). In some experiments, images of adherent cells were obtained after crystal violet staining and before solubilization, using an IX70 inverted microscope (Olympus) equipped with a Micropublisher 3.3 RTV digital camera (Q Imaging).

### Surface plasmon resonance

Kinetic studies of integrin–ligand interactions were performed using a BIAcore T200 instrument (Cytiva). Ligands (S1-RBD or FNIII10) were immobilized using amine-coupling chemistry according to the manufacturer’s instructions (BR-1000-50). Briefly, ligands diluted in 10 mM sodium acetate (pH 4.0, Cytiva) were immobilized on an EDC/NHS-activated CM5 chip (Cytiva) to a target level of 800 to 1000 RU. Excess amine-reactive groups were inactivated with 1 M ethanolamine (pH 8.5, Cytiva). Immobilization buffer was 10 mM Hepes buffer pH 7.4 containing 0.05% n-octyl-β-D-glucopyranoside (OGPS, Anatrace), 150 mM NaCl_2_, 2 mM MnCl_2_, 2 mM MgCl_2_, and 0.2 mM CaCl_2_. Lyophilized integrins were reconstituted with 50 mM Tris, pH 7.4 containing 25 mM OGPS, 1 mM DTT, 150 mM NaCl_2_, and divalent cations (α_v_ integrins: 2 mM MnCl_2_, 2 mM MgCl_2_, and 0.5 mM CaCl_2_; α_5_β_1_ integrin: 2 mM MnCl_2_). Double-referenced binding experiments were performed in parallel flow cells for S1-RBD and the corresponding positive control (FNIII10 for α_v_ integrins; FNIII8-10 for α_5_β_1_) (46) using a flow rate of 30 μl/min for 1 min with a dissociation time of 5 min. Surfaces were regenerated between injections using two 30-s injections of 20 mM EDTA and 1 M NaCl (47). Kinetic parameters were determined by fitting a 1:1 binding model with globally fit parameters for each collected data set using Biacore T200 Evaluation software (Version 3.2, GE). Owing to the large difference in dissociation rates between the two ligands, only the first 10 s of the dissociation curves were considered for S1-RBD data sets. Quality of fit was determined by agreement between measured and calculated Rmax and chi-squared values. Data sets not producing high-quality kinetic fit were excluded from calculation of kinetic parameters.

### Immunofluorescence microscopy

Acid-washed glass coverslips were coated with saturating concentrations of protein (500 nM; S1-RBD or FNIII10) for 1 h at 37 °C. FN-null MEFs (2.5 × 10^3^ cells/cm^2^) were seeded in AimV/SF media and incubated at 37 °C, 8% CO_2_ for 4 h. Cells were then fixed with 2% paraformaldehyde in PBS and processed for immunofluorescence microscopy as described (107). Cells were incubated with primary antibodies or TRITC-phalloidin diluted in PBS containing 0.1% Tween 20, 1% BSA, and 1 mM phenylmethylsulfonyl fluoride for 1 h at room temperature. Bound antibodies were detected with Alexa^448^-, Alexa^549^-, or Alexa^647^-labeled goat anti-rabbit or -mouse secondary antibodies and visualized using a BX60 fluorescence microscope (Olympus) equipped with an epifluorescent lamp (Lumen Dynamics) and an EXi Blue Fluorescence Camera (Q Imaging), acquired with QCapture software.

### Immunoblot analysis

FN-null MEFs (3.4 × 10^4^ cells/cm^2^) or SAECs (6.7 × 10^4^ cells/cm^2^) were seeded on wells precoated with saturating concentrations of S1-RBD (500 nM), FNIII10 (500 nM), or fibronectin (10 μg/ml) and incubated at 37 °C for 1 h (FN-null MEFs) or 4 h (SAECs). Cells were lysed with 40 μl/cm^2^ SDS-RIPA buffer (50 mM Tris, 150 mM NaCl, 1 mM EDTA, 1% Triton X-100, 0.1% sodium dodecyl sulfate, 0.5% sodium deoxycholate, pH 7.6) containing 1 mM sodium orthovanadate, 1 mM phenylmethylsulfonyl fluoride, and 1× protease inhibitor cocktail (Sigma S8830). Cell lysates were analyzed by SDS-PAGE and immunoblotting (108). Immunoblots were blocked with either 5% nonfat milk or 3% BSA in Tris-buffered saline containing 0.1% Tween 20 (TBS-T). Membranes were incubated overnight at 4 °C with primary antibodies diluted in TBS-T. Vinculin was used as the protein loading control. Blots were then washed with TBS-T, incubated with horseradish peroxidase–conjugated secondary antibodies, and developed using SuperSignal West Pico Chemiluminescent Substrate (Thermo Scientific). Blots were imaged using a ChemiDoc imaging system (Bio-Rad).

### Bead binding assay

Fc-RBD or mouse IgG (667 nM in PBS) was immobilized on Protein G fluorescent particles (5.0–5.9 μm diameter) according to the manufacturer’s instructions. Unbound protein G sites were blocked with 3% BSA. Biotin-labeled 20-mer peptides encompassing the RGD region of SARS-CoV-2 or corresponding KGD region of SARS-CoV-1 were immobilized on streptavidin fluorescent particles (5.0–7.9 μm diameter) according to the manufacturer’s instructions and blocked with 1% BSA. Ligand-bound beads were washed and resuspended in small airway epithelial media with or without 1 mM MnCl_2_ immediately prior to use. Glass coverslips were coated with 10 μg/ml laminin, and SAECs were seeded at a density of 5.4 × 10^4^ cells/cm^2^. SAECs were allowed to adhere overnight in growth media and washed twice with basal media immediately prior to bead treatment. SAEC monolayers were incubated with 1 × 10^6^ beads/cm^2^ beads for 2 h at 4 °C. Assays were performed at 4 °C to minimize nonspecific endocytosis of particles. Unbound beads were removed by gentle washing with basal media, and cells were fixed with 1% paraformaldehyde in PBS. hSAECs monolayers were visualized by low-power phase microscopy. Cell-bound beads were detected by fluorescence microscopy and counted using FIJI software (NIH, cell counter plugin). The extent of bead binding in each condition was quantified as the mean number of fluorescent beads in 3 to 6 independent regions of interest (0.6 mm^2^) per well.

### Statistical analysis

Data are presented as mean ± standard error unless otherwise stated. Experiments were performed in duplicate or triplicate on a minimum of two independent days. All statistical analyses were performed using GraphPad Prism (version 9). Statistical differences between groups were identified by two-tailed *t* tests or one- and two-way ANOVAs as indicated, using Bonferroni’s posttest and a *p*-value threshold < 0.05.

## Data availability

All data are contained within the article, with supplementary data available upon request (denise_hocking@urmc.rochester.edu).

## Conflict of interest

The authors declare that they have no conflicts of interest with the contents of this article.
